# Physiologically Based Pharmacokinetic Modelling of Hydroxyurea in Patients with Sickle Cell Disease: A Special Focus on Lactating Women and Breastfed Infants to Inform Safe Dosing and Breastfeeding Strategies

**DOI:** 10.3390/ph19020220

**Published:** 2026-01-27

**Authors:** Khaled Abduljalil, Neel Deferm, Anna Murphy, Iain Gardner

**Affiliations:** 1 Certara Predictive Technologies, Level 2-Acero, 1 Concourse Way, Sheffield S1 2BJ, UK; neel.deferm@certara.com (N.D.); anna.murphy@certara.com (A.M.); iain.gardner@certara.com (I.G.); 2 Pharmides BV, 3900 Pelt, Belgium

**Keywords:** hydroxyurea, PBPK, pharmacokinetics, sickle cell disease, lactation, neonates, paediatric

## Abstract

**Background/Objectives**: Hydroxyurea is currently the standard disease-modifying therapy for reducing sickle cell disease (SCD) complications; however, drug labels currently advise discontinuation of breastfeeding during hydroxyurea therapy due to limited human data on the risk of hydroxyurea exposure in breastfed neonates. **Methods**: A physiologically based pharmacokinetic (PBPK) model for hydroxyurea was built and verified with data from non-lactating adult patients with SCD. The model was then extended to predict hydroxyurea in nursing and in paediatric populations. Predictions were compared to the observed data. **Results**: The PBPK model predictions for hydroxyurea pharmacokinetics described the observed data in both adult and paediatric subjects with SCD. Observed concentration profiles were within the 5th–95th prediction intervals, and predicted PK parameters were within 2-fold of the observed values. The predicted milk-to-plasma ratio was 0.8. Neonatal exposure to hydroxyurea via breast milk as a percentage of maternal exposure increased from 0.6% at 1 day to 10% at the 4th week postpartum before declining to 5%, 3%, and 2% at 6, 9, and 12 months postpartum, respectively. **Conclusions**: About 56% of total milk hydroxyurea exposure is within the first 3 h of post-maternal dose. Disposal of this early milk would reduce the exposure of breastfed children. The reduction in exposure is especially pronounced around the first 1 month postpartum. Lactation PBPK models offer a physiological approach to assess real-life scenarios that are difficult to investigate in clinical studies and provide useful results for future clinical study design and clinical recommendations. This was exemplified with hydroxyurea in the current work.

## 1. Introduction

Sickle cell disease (SCD) is a group of inherited red blood cell disorders affecting the β-globin gene, resulting in the polymerisation of haemoglobin and subsequent changes in red blood cell morphology. Sickle cell anaemia (SCA) is the most severe form of SCD and causes haemolysis, pain crises, and chronic organ damage [1]. As of 2021, SCD is estimated to affect about 7.7 million people worldwide, directly causing an estimated 34,000 annual deaths. More than 70% of SCD cases are in sub-Saharan Africa, but SCD also occurs to a lesser degree among people in India, the Middle East, Brazil, and southern Europe [2]. It affects nearly 100,000 individuals in the United States [3].

While there is no universal cure for SCD, major advances have made disease management, focusing on relieving symptoms, preventing complications, and improving quality of life, more effective [4]. Hydroxyurea (also called hydroxycarbamide) is a cornerstone in SCD pharmacotherapy with the main therapeutic benefit appearing to come from increased foetal haemoglobin (HbF) production, which inhibits haemoglobin polymerisation [5], thereby reducing red blood cell sickling and the risk of blood vessel blockage [6,7]. Regular hydroxyurea use reduces the frequency and severity of SCD complications, including painful crises, vaso-occlusion, and acute chest syndrome. It also improves blood flow and oxygen delivery throughout the body, reducing the need for blood transfusions, and lowers mortality in patients with severe SCA [8,9]. Long-term use of hydroxyurea has been shown to be associated with increased survival [10].

The pharmacokinetics (PK) of hydroxyurea in adult patients have been evaluated in many clinical studies; however, the only available data for oral hydroxyurea bioavailability comes from investigations in adult cancer patients and was found to be 79% to 108% [11,12]. While hydroxyurea might exhibit nonlinear PK after administration of relatively high doses (~50 to 550 mg/kg/day) for cytotoxic indications [12,13], its PK in SCD adult patients with normal renal function after oral administration is linear at the usual daily dose of 15–35 mg/kg [14]. A study in HIV patients with normal renal function also showed linear PK after oral administration of 500 mg twice daily for 4 weeks [15]. Hydroxyurea reaches peak plasma concentrations in 1 to 4 h following oral administration. It distributes throughout the body with an apparent volume of distribution close to that of body water, with approximately 35% to 40% of the orally administered dose being excreted through urine in SCD patients with apparently normal renal function [14,16]. Hydroxyurea PK in infants is similar to that observed in older children and adults, with body weight alone accounting for much of the interpatient variability across the population [17,18]. Weight-based dosing results in similar systemic exposures across different age ranges (0.5 to 18 years), suggesting that any additional age-related dosage adjustments are not necessary [17]. In a comparative study, hydroxyurea PK parameters obtained from different paediatric clinical trials (total of 451 SCA patients aged 0.5–19.5 years) conducted in different regions (USA, Uganda, Kenya, and Jamaica) showed that children from Africa had slightly lower volumes of distribution, but absorption rate and clearance were similar across regions [19]. Rodent data indicated that hydroxyurea metabolism involves degradation by urease (~80% of total metabolism) to urea and oxidation (~10%) to produce nitric oxide (see [20]) with another minor pathway found in intestinal bacteria, but the mechanism of this catabolism has not yet been elucidated. Data from rodent liver microsomes showed that cytochrome P450 enzymes are involved in hydroxyurea metabolism at high concentrations (25 mM) [21],. However, a study using human liver microsomes showed that hydroxyurea is neither a substrate nor an inhibitor of cytochrome P450 enzymes [22]. The latter study also showed that the ATPase activity in the absence of verapamil was not increased by hydroxyurea, suggesting that hydroxyurea is not transported by P-glycoprotein [22].

Hydroxyurea is available as oral capsules and tablets and was approved in 1998 for treatment of SCD in adult patients [23,24]. In December 2017, regulatory approval was issued for paediatric patients with SCA as young as 2 years old [25]. In 2024, an oral solution (XROMI^®^) was approved for treating SCD in infants from the age of 9 months [26]. For both adult and paediatric patients with SCD, hydroxyurea is usually prescribed at an initial starting oral dose of 15–20 mg/kg once a day and is then gradually increased to a maximum daily dose of 35 mg/kg (~2000 mg), with dose escalation stopping when either a clinical benefit (i.e., increased HbF) or toxicity, such as myelosuppression, occurs [27,28].

Current hydroxyurea labels urge nursing mothers with SCD to cease breastfeeding when using hydroxyurea, even though hydroxyurea is excreted into breast milk at seemingly low amounts according to earlier case report studies in a nursing mother with leukaemia [29] and in another nursing mother with SCA [30]. Moreover, a clinical hydroxyurea PK study in 16 lactating mothers (2–22 months postpartum) reported a hydroxyurea relative infant dose (RID) of 3.4%, calculated from the estimated daily infant’s dose (DID) of 0.46 ± 0.16 mg/infant kg and a constant milk intake of 150 mL/kg/day [31]. Since the estimated RID for hydroxyurea in that clinical study is below the 10% recommended safety threshold and the DID is below the 20–30 mg/kg/day doses that are currently used to treat young infants with SCA, that clinical study suggested that breastfeeding should not be contraindicated for women receiving hydroxyurea for SCD treatment [31]. The 10% guideline is a general index [32], and for cytotoxic compounds like hydroxyurea, this 10% cut-off value may not be appropriate.

Since the doses administered in these lactation studies are at the lower end of the clinical dose escalation range (~15–20 mg/kg) and the assumed daily milk intake is constant, it would be informative to predict the milk exposure and assess the expected RID and DID for the range of clinically used hydroxyurea doses, as well as to assess the exposure in breastfed neonates and young infants, who have immature renal function and consume varying amounts of breast milk.

Physiologically based pharmacokinetic (PBPK) modelling enables the integration of known postpartum physiological changes that influence maternal PK [33], as well as lactation models that estimate drug transfer into breast milk [34]. Additionally, PBPK models can be developed for nursing infants to account for the immaturity of drug elimination pathways and other developmental factors that influence drug disposition [34]. An earlier paper reviewed current knowledge gaps in SCD treatment and potential benefits that can be achieved from future development of a hydroxyurea PBPK model [35]. The approach was then applied for dose assessment in SCD patients with renal impairment [36]. In this work, a PBPK model was extended to predict the excretion of hydroxyurea into breast milk. This hydroxyurea PBPK model can be used to supplement available clinical lactation data and drug labels on hydroxyurea by providing more comprehensive information regarding infant drug exposures during breastfeeding. Consequently, the objective of this study was to develop PBPK models for hydroxyurea in both lactating mothers and nursing infants to predict infant exposure based on the estimated infant dose to optimise dosing (and breastfeeding) strategies that maintain maternal therapeutic efficacy while minimising toxicity risks for the infant.

## 2. Results

The final parameter inputs for the hydroxyurea PBPK model are given in Table 1 and collected clinical trials that investigate hydroxyurea PK are given in Table 2.

**Figure 1:**
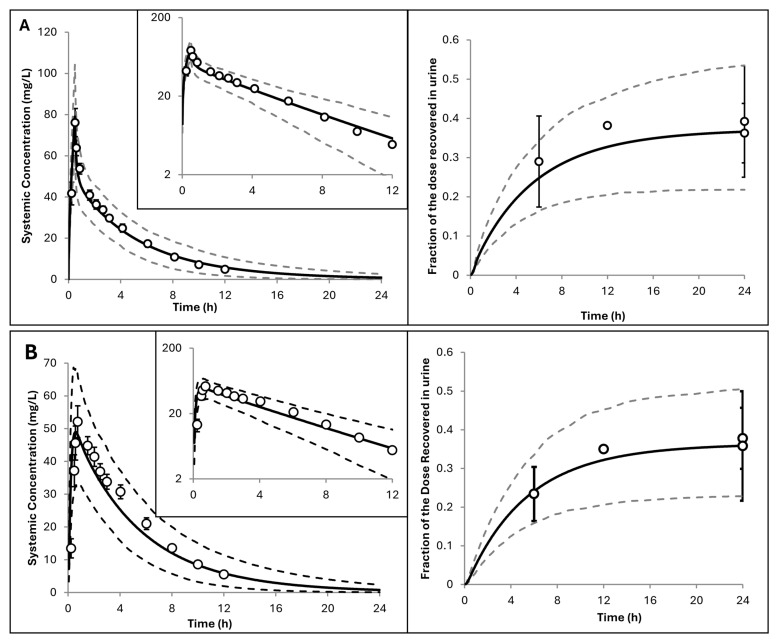
Simulated versus observed hydroxyurea plasma concentrations and 24 h urinary excretion as a fraction of the dose in adult cancer patients ((**A**) intravenous and (**B**) oral administration) (see Table 2). Solid lines represent predicted mean values; dotted lines show the 5th and 95th percentiles. Open circles indicate observed data [11] with standard deviations (error bars). Inserts use a logarithmic scale.

A PBPK model for hydroxyurea in adult subjects using the cancer population was constructed. The observed data reported in cancer patients [11] was used to optimise some parameters (Figure 1 and Table 3). The intravenous data was used to optimise the Kp scalar and the balance of renal and metabolic (additional) clearance used in the PBPK model. The PBPK model accurately predicted the mean plasma concentrations, the terminal phase of the plasma concentration vs. time profile (Figure 1A), and the urinary excretion profiles (Figure 1B). For oral data, refinement of an intestinal apical uptake process (CL_int,T_) was required to accurately describe the absorption phase. Once the model was parameterised and verified against data from [11], it was used without further modification to simulate a range of clinical studies where observed data in adults is reported in the literature. Predicted PK parameters also fell within a 2-fold range of observed values, supporting the overall adequacy of the model and its parameter optimisation (Table 3).

**Figure 2:**
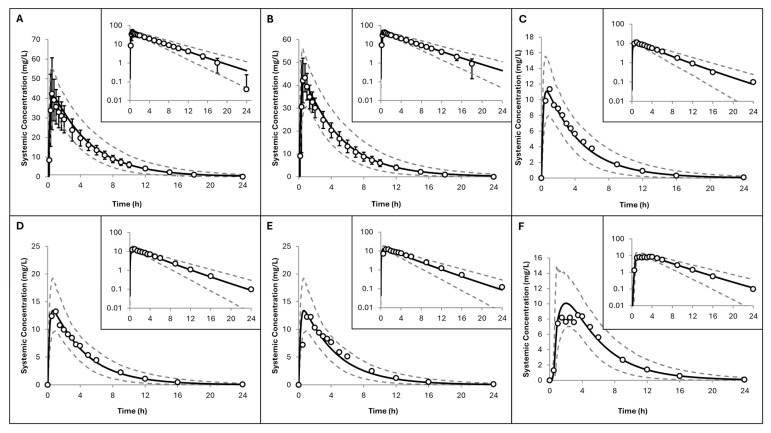
Simulated versus observed hydroxyurea plasma concentrations in adult healthy volunteers across six clinical trials: (**A**,**B**) [41], (**C**–**F**) [42]. Trial designs are summarised in Table 2. Solid lines represent predicted mean values; dotted lines show the 5th and 95th percentiles. Open circles indicate observed data with standard deviations (error bars, where available). Inserts use a logarithmic scale.

In healthy volunteers (Figure 2), the PBPK model also demonstrated good agreement with observed plasma concentration profiles of oral hydroxyurea under both fasted (Figure 2A–E) and fed (Figure 2F) conditions. Predicted PK parameters across these trials were within 1.5-fold of observed values (Table 3), confirming the adequacy of the baseline model.

**Figure 3:**
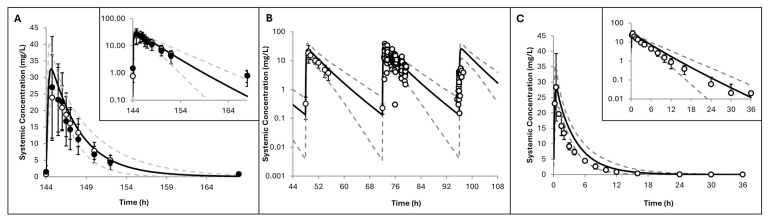
Simulated versus observed hydroxyurea plasma concentrations in adult patients with SCD across three clinical trials. (**A**) Black dots represent tablets; white dots represent capsules [43]; (**B**) [16] and (**C**) [14]. Trial designs are summarised in Table 2. Solid lines represent predicted mean values; dotted lines show the 5th and 95th percentiles. Circles indicate observed data with standard deviations (error bars, where available). Inserts display data on a logarithmic scale.

In adult patients with SCA, simulated hydroxyurea plasma concentrations also aligned well with observed data (Figure 3). The model slightly overpredicted concentrations in one study [14] (Figure 3C), reflected by a P/O ratio for AUC_inf_ of 1.5 and CL_po_ of 0.72. Despite this, the P/O ratios for PK parameters remained within 2-fold error margin, indicating that the model’s performance in the adult SCA population is acceptable (Table 3).

**Figure 4:**
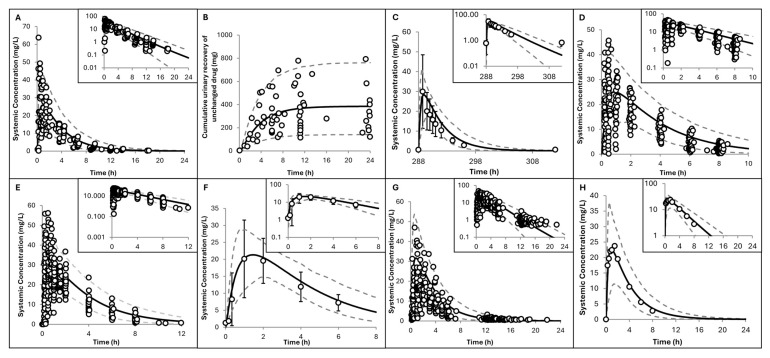
Simulated versus observed hydroxyurea (**A**,**C**–**H**) plasma concentrations and (**B**) cumulative urinary recovery of unchanged drug in paediatric patients with SCD across seven clinical trials: (**A**,**B**) [18], (**C**) [43], (**D**) [44], (**E**) [45], (**F**) [46], (**G**) [17], (**H**) [47]. Trial designs are summarised in Table 2. Solid lines represent predicted mean values; dotted lines show the 5th and 95th percentiles. Circles indicate observed data with standard deviations represented as error bars when these were reported and individual data were not available. Inserts display data on a logarithmic scale.

Simulations in paediatric SCA patients are shown in Figure 4, with predicted versus observed plasma concentrations (Plots A, C–H) and cumulative urinary recovery (Plot B) of hydroxyurea. Predicted mean values closely matched the observed data, and the prediction intervals adequately captured the observed variability. As shown in Table 4, the P/O ratios for all PK parameters across the clinical studies were within the pre-defined acceptable range of 2-fold error, supporting the model’s robustness.

**Figure 5:**
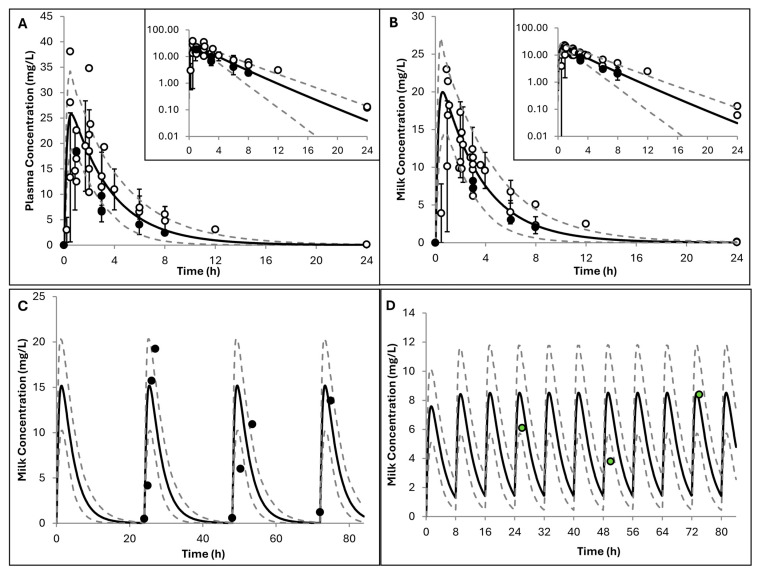
Simulated versus observed hydroxyurea concentrations in lactating women. (**A**) Plasma and (**B**) milk concentrations with black and white dots represent observations in sickle cell disease and healthy nursing mothers, respectively [31]. (**C**,**D**) Hydroxyurea in breast milk from a lactating mother with sickle cell disease [30] and from another lactating woman with leukaemia [29]. Trial designs are summarised in Table 2. Solid lines represent predicted mean values, and dotted lines show the 5th and 95th percentiles from 200 virtual individuals. Circles indicate observed data with error bars, where available, represent the standard deviations. Inserts display data on a logarithmic scale.

Simulations for lactating women showed good agreement with observed plasma and milk concentrations after single and multiple doses (Figure 5). In addition, predicted PK parameters in healthy lactating individuals were consistently within a 2-fold error range (Table 3), supporting the robustness of the model. The predicted M/P ratio by the developed hydroxyurea PBPK model was 0.77 ± 0.05 (mean ± SD), which was consistent with observed values of 0.88 (arithmetic mean) and 0.83 (geometric mean) ± 0.26 (SD) [31]. Results for lactation parameters obtained upon replicating Ware et al.’s study [31] are shown in Figure A2 in Appendix A.

Table 5 shows the maternal exposure, predicted M/P, DID, and RID of hydroxyurea in lactating women receiving either 15 mg/kg/day (low dose) or 35 mg/kg/day (high dose) at different postnatal times. Since the PBPK model is linear, the 2.3-fold increase in the dose was associated with a similar increase in maternal AUC_tau_ (35 mg/kg/day vs. 15 mg/kg/day: 220 vs. 94 h·mg/L at day 1; 240 vs. 103 h·mg/L at 12 months).

**Figure 6:**
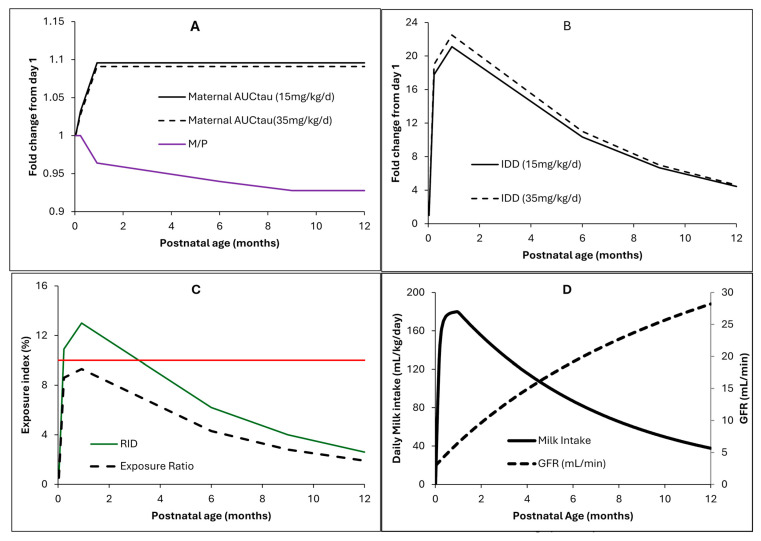
Hydroxyurea PBPK model application results. (**A**,**B**) Relative changes in hydroxyurea lactation parameters with postpartum time shown as fold changes relative to day 1 for the low (15 mg/kg) and high (35 mg/kg) daily doses. (**C**) Comparison of the RID vs. exposure ratio with the horizontal line representing the 10% threshold. (**D**) Changes in neonatal GFR and daily milk intake.

The predicted fold change in DID mirrors these changes with baseline at birth of 0.2 ± 0.1 mg/kg/day for the high dose vs. 0.09 ± 0.03 mg/kg/day for the low dose. From day 1 through the end of the first year postpartum, maternal hydroxyurea plasma exposure showed little to no increase, with only a minimal decrease in the M/P ratio; both DID and RID profiles changed with postpartum time (Table 5 and Figure 6), coinciding with the physiological change in infant daily milk intake [33]. The highest nominal DID was 1.9 ± 0.6 mg/kg/day and 4.5 ± 1.4 mg/kg/day for the low and high doses, respectively (Table 5). Infant hydroxyurea exposure was about 10% of maternal exposure at 1 month postpartum and varied in parallel with changes in the RID (Table 5).

## 3. Discussion

In the current study, a hydroxyurea PBPK model that accurately predicted hydroxyurea PK in adults, including nursing mothers, and paediatric subjects was developed. Hydroxyurea is currently the first-line disease-modifying treatment for SCD, with clear therapeutic benefit [27,28]. However, based on evidence of teratogenic effects in animal data and scant observations regarding transfer of hydroxyurea into breast milk, nursing women are advised to stop taking hydroxyurea during breastfeeding or stop nursing (Hydrea^®^ [23] and Siklos^®^ [24]). In this work, we assessed the magnitude of hydroxyurea exposure at clinically used low and high doses in non-lactating adult and paediatric patients with SCD. In addition, we included simulations in neonates and infants dosed with hydroxyurea to address key knowledge gaps as these designs have not been evaluated in clinical settings. Since SCD is heterogenous and published clinical studies reported their results for the mixed variants, mainly HbSS and HbS/β-thalassemia, the current study mimics the original clinical studies, and hence the developed PBPK model and obtained results do not differentiate between the different variants.

The hydroxyurea PBPK model was first developed using non-lactating, non-SCD populations to ensure that the hydroxyurea compound file was adequately parameterised. The model was able to adequately describe data from multiple populations. The current model includes a non-specific linear systemic clearance pathway due to a lack of knowledge of specific metabolic enzymes involved in hydroxyurea clearance, and a linear renal filtration component. In the paediatric model, systemic clearance is scaled using allometric principles with a coefficient of 0.75, while renal clearance is scaled according to age-related maturation of the GFR [48]. It is likely that renal and liver transporters are involved in hydroxyurea disposition; however, these active processes have not been quantified yet and so could not be accounted for mechanistically in the model [49]. Hydroxyurea is assumed to undergo passive absorption, represented by P_trans,0_, but this underpredicts observed absorption; therefore, an intestinal transporter was incorporated. Several of the transporters associated with hydroxyurea are expressed in the human intestine [49]; however, the kinetics of the main key transporter(s) have not been quantified yet. Thus, the model assumes a generic uptake transporter in the gut. Due to absence of information, the intrinsic clearance and expression of this transporter were assumed to be constant across all the simulated populations, including the paediatric population.

**Figure A1:**
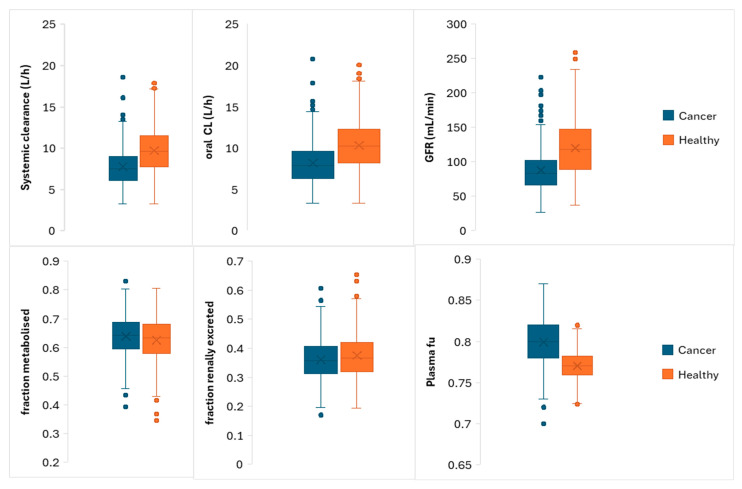
Whisker boxplots of hydroxyurea model parameters in healthy and cancer populations.

Since there are no drug–hydroxyurea interaction studies examining these active transport processes, the model assumptions for transporter kinetics remain uncertain. In addition, the only available intravenous hydroxyurea data used to optimise distribution and clearance in the model came from cancer patients. Cancer patients tend to have lower renal function compared to healthy volunteers [50]; hence, the cancer population within the Simulator was used. A comparison between hydroxyurea parameters in healthy and cancer populations is given in Appendix A (Figure A1). It is best practice in PBPK to use a population with a physiology that is close to the population from which the PK data were derived to remove the impact of this covariate. This is a fundamental step in in vivo extrapolation [51]. Therefore, compound input parameters in Table 1 represent typical values in normal healthy adult individuals. When using the paediatric population, for example, these input parameters are then scaled specifically for the paediatric population according to the age of the child. While to date there are no comparative clinical studies demonstrating to what degree cancer can modify hydroxyurea PK relative to healthy volunteers, Table 2 shows that the observed average clearance in cancer (7.5 L/h [11]) was lower than the clearance in healthy volunteers (9.3 L/h [41]) after oral administration of 2 g to both groups, and the exposure in healthy volunteers was 71% of that in the cancer population for the same dose. The ability of the model to replicate PK in SCD using data from individuals with cancer [11] suggested the suitability of that dataset for model development. Figure 1 and Figure 2 (and Table 3) show that the healthy volunteer PBPK model using these baseline parameters performed well in describing the oral PK data in healthy individuals. Likewise, predictions using this baseline model without modification predicted SCD PK within 1.5-fold of observed data (Table 3). Table 2 also shows that the observed (and the predicted) clearance in healthy subjects [41,42] is similar to that in SCD patients [16,43], which suggests that the overall net effect of pathophysiological changes in SCD did not affect hydroxyurea clearance. Further, recovering hydroxyurea concentration profiles in SCD patients using healthy volunteers’ physiology re-affirms the assumption that hydroxyurea PK is comparable in healthy and SCD patients. However, a caveat is that one cannot generalise these results to other compounds with different physicochemical and PK characteristics such as high binding to protein, higher lipophilicity, and different absorption and disposition profiles. The observed SCD data used for verification was from SCD patients with confirmed normal renal function, whereas there is a high prevalence of structural and functional kidney abnormalities in SCD patients because of disease progression, which can result in renal impairment [52]. Functional abnormalities include both hyperfiltration and reduced glomerular filtration, leading to large variability in renal function in SCD patients [16]. However, longitudinal changes in renal function with treatment have not been incorporated in the current model.

The lactation PBPK model in heathy volunteers performed well at predicting both maternal and milk hydroxyurea concentrations (Figure 5) reported from various clinical studies [30,31]. The observed plasma AUC_0–24_ in 14 healthy lactating individuals showed high variability (ranged from 37 to 132.6 h·mg/L). The two nursing patients with SCA had plasma AUC_0–24_ values of 26.5 and 59.2 h·mg/L; the latter falls within the broader healthy range, suggesting broadly comparable systemic exposure between the two groups [31]. Furthermore, the M/P ratio predicted by the hydroxyurea PBPK model was 0.77 ± 0.05, which matched the observed ratio of 0.88 ± 0.30 (and closely matched the observed geometric mean of 0.83) [31]. The interindividual variability in physiological parameters and their changes from birth until 12 months of postpartum (as has been described in details in our earlier work [33]) included in the model did not fully explain the observed variability in the M/P ratio, as the model does not account for other potential sources of variability such as any effects of the disease itself on milk composition, milk pH, and breastfeeding frequency.

**Figure A2:**
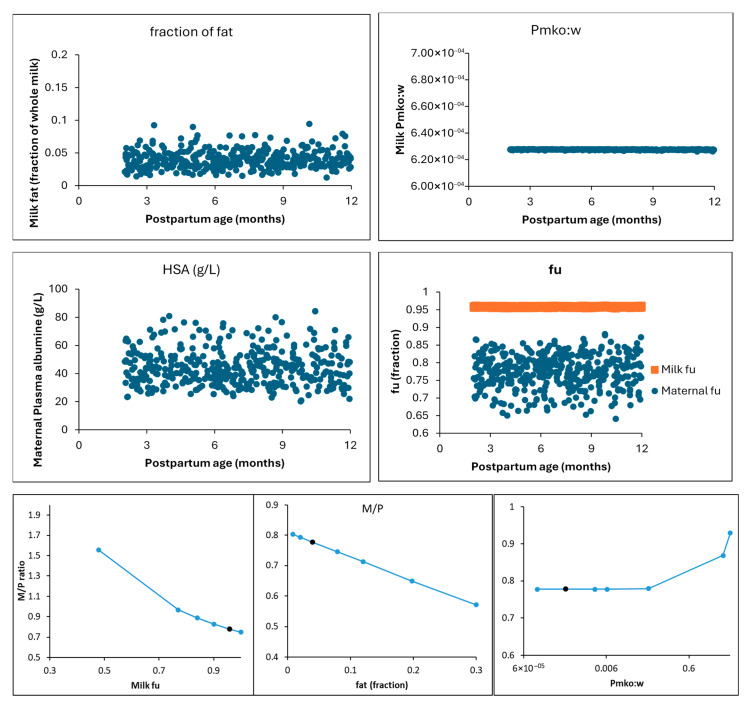
Hydroxyurea lactation model parameters during 2 to 12 months of postpartum after Ware et al.’s [31] study design. The lower three figures are model sensitivity to milk fu, fat fraction, and estimated Pmko:w (i.e., Papp_mk_). Black circles show the model inputs predicted from milk and the drug attributes (see Section 4).

The M/P ratio of hydroxyurea, a hydrophilic compound, is not sensitive to the fat fraction (f_fat_) in milk over the range of physiologically realistic values (see Figure A2 in Appendix A). Again, the model is sensitive to the apparent milk fat-to-water partition (Papp_mk_) parameter only if hydroxyurea is lipophilic with LogP_o:w_ ≥ 1.2, which is not the case for hydroxyurea (LogP_o:w_ of −1.8) (Table 1). Milk fu was predicted from maternal plasma fu according to Atkinson et al. [40], as the original study [31] did not report the milk fu for hydroxyurea. Since approximately 80% of hydroxyurea is free in plasma, it is expected to be almost free in milk, as milk albumin is only 10% of plasma [40]. In contrast, sensitivity analysis showed that hydroxyurea M/P is sensitive only if the milk fu is less than plasma fu. It is still unknown whether hydroxyurea has any affinity towards milk whey protein, but sensitivity analysis (Figure A2) shows that the milk fu only needs to change minimally from the predicted value of 0.96 to a value of 0.90 to match the observed geometric (and to 0.85 to match the arithmetic) mean M/P ratio. It is worth mentioning that when the milk parameter was forced to fit the observed M/P ratio (0.88), compared to using the predicted M/P value of 0.77, the expected increase in RID and DID was less than 15%; hence, the predicted milk fu value was kept in the model.

Table 5 shows the model predicted postpartum-dependent RID, summarising how changes in maternal physiology and milk composition over the first year postpartum influence infant exposure [33]. RID was predicted to be dose-independent across the range of doses used to treat SCD. Notably, the reported RID of 3.4% from a PK study of mothers who had been lactating for a mean of 9 months [31] aligned closely with the model’s prediction of 4.0% (Table 5). The highest predicted RID occurred at the end of the first month postpartum, coinciding with peak infant milk intake (Figure 6). Across postpartum periods, the predicted exposure ratios followed the same pattern as the RID but were generally slightly lower. Unlike RID, the exposure ratio accounts for physiological changes occurring in the breastfed infant (such as growth and maturation), hence offering a more realistic metric of assessment in lactation studies. Prior knowledge of the drug exposure in children, which can be taken from the paediatric PBPK model results, is required for applying the exposure ratio, similar to what has been performed in the current work for hydroxyurea. Utility of this index over the RID requires further evaluation with different drugs, where slow maturation processes, i.e., slow ontogeny, are key determinants of the PK in neonates and infants.

The drug label does not yet address the safety or risk of hydroxyurea exposure in breastfed infants younger than 6 months. Simulated DIDs, RIDs, and exposure levels for neonates aged 0.033 to 0.92 months indicate that, immediately after birth, the RID is only 0.6% (Table 5), and the neonate’s exposure represents <2% of the exposure observed in 9-month-old SCD children treated with hydroxyurea. Although renal function is immature in neonates, the overall hydroxyurea exposure is minimal immediately after birth because the daily milk intake is very low. Indeed, the average milk consumption increases from 9 mL/kg/day at 0.033 months to 180 mL/kg/day at 0.92 months. Since infants with SCA may receive therapeutic doses of 20 mg/kg/day at ages as early as 5 months to maximise opportunities for organ injury prevention (see discussion in [27]), the developed model suggests that any additional exposure from breast milk at this age is small (~5% of the therapeutic dose), indicating a low risk for breastfed infants. While this interpretation is based on PK and exposure, neonatal sensitivity toward hydroxyurea’s adverse effects may or may not be different, as safety data are limited [27], so additional evidence is needed to confirm safety. Moreover, our results also highlight that hydroxyurea exposure around 1 month postpartum may be relatively higher than in younger neonates and older infants, due to peak milk intake combined with still-maturing renal function (Figure 6). In older infants, on the other hand, exposure decreases as milk intake falls and renal function matures.

Taken together, our results suggest that breastfeeding does not need to be discontinued whilst taking hydroxyurea. Instead, infant exposure can be safely minimised through appropriate risk management strategies informed by hydroxyurea PK. As shown in Table 5, hydroxyurea concentrations in breast milk peak within the first 3 h (AUC_3h_) after maternal dosing, contributing to approximately 56% of the total milk exposure. The estimated changes in the DID upon pumping-and-dumping the collected milk after 2, 3, and 4 h of maternal intake of hydroxyurea are given in Appendix A (Table A2). Consequently, practical measures such as avoiding breastfeeding (collecting and discarding breast milk) during the first 3 h post-dose, substituting with expressed breast milk collected prior to dosing, or temporarily using an alternative feeding source can substantially reduce infant exposure while maintaining the therapeutic hydroxyurea effect for the mother. For older infants (6–12 months), hydroxyurea intake via breast milk is extremely low and represents only a small fraction of the standard therapeutic dose.

Finally, it is still uncertain to what degree the use of a healthy population within the PBPK model can represent the SCD population. This gap, which is a limitation of this work, can only be addressed by developing SCD-specific paediatric and adult populations that include pathophysiological changes that can affect PK parameters, such as metabolising enzyme and transporter expression, haematocrit, tissue blood flow, and changes in renal and liver function in different stages of SCD. While, from the obtained results, the impact of pathophysiological changes appears to be small for hydroxyurea, these changes cannot be generalised for other drugs, especially those with lower solubility and higher lipophilicity or those highly bound to plasma protein. The assumption of full maturation of the gut transporter in children, i.e., no ontogeny in protein activity per mg of protein, is still uncertain. Sensitivity analyses in adult and infant populations are given in Appendix A (Table A1), indicating that the impact of changes in the activity of this transporter is less in infants compared with adult population due to the small intestinal absorptive surface area in infants. Conflicting data on changes in albumin levels are available in SCD patients. In a large-sample-size study of 1933 adults with SCD, serum albumin was at 43 (range: 39–46) g/L [53], which is similar to the levels in healthy subjects; others reported a 20% reduction in serum albumin in 54 adults with SCA (35 ± 8 g/L) compared with a control group of 30 individuals (44 ± 3 g/L) [54]. However, hydroxyurea has a plasma fu of 0.8, and a minimal reduction in plasma albumin would not result in a dramatic increase in the clearance of hydroxyurea in adult patients with SCA. The magnitude of SCD impact on albumin is expected to be attenuated in paediatric patients where the plasma albumin level is lower (63%, 87%, and 92% of adult magnitude at birth, 1 month, and 6 months, respectively) [48]. The developed model does not account for the nonlinearity observed at high doses (~50 to 550 mg/kg/day) for cytotoxic indications [12,13].

## 4. Materials and Methods

### 4.1. Software

Data analysis was conducted using Microsoft Excel 2016 (Microsoft Corporation, Microsoft Office Professional Plus 2016, https://products.office.com (accessed on 27 November 2025). Numerical data were extracted from published figures using GetData Graph Digitizer (version 2.26.0.20). All hydroxyurea PK predictions across different populations were performed using the Simcyp Simulator V24 (Certara Predictive Technologies, Sheffield, UK).

### 4.2. Workflow

The hydroxyurea PBPK model was developed using a stepwise approach. First, the model was developed to describe the data observed in the adult population. The model’s performance was then verified with additional data from healthy and SCD adult patients. Second, the adult PBPK model was extrapolated to predict hydroxyurea in paediatric patients with SCD. Likewise, the adult PBPK model was extrapolated to predict hydroxyurea in nursing mothers by including the lactation model that accounts for milk and maternal attributes. The developed model was finally executed for multiple real scenarios to assess the impact of different administered hydroxyurea doses and lactation periods on neonatal exposure to hydroxyurea via maternal milk.

All PBPK model simulations consisted of 20 trials, with 20 virtual subjects per trial, to ensure adequate characterisation of the predicted PK parameters and their variability. In all cases, the demographics (age, sex, and weight) of the virtual population were set to match those reported in the corresponding clinical studies.

### 4.3. Model Building

First, physicochemical properties and plasma binding information published for hydroxyurea were acquired from public domain sources. Disposition and absorption parameters of hydroxyurea were then added stepwise into the model. Tissue distribution of hydroxyurea was modelled using a full-body PBPK model, with tissue partition coefficients (Kp) predicted according to the method of Rodgers and Rowland [39]. Elimination of hydroxyurea was modelled as passive renal filtration with renal clearance (CL_r_). Both the global tissue scalar and the CL_r_ were optimised to describe observed hydroxyurea PK data following intravenous dosing in adult patients with cancer [11], as equivalent data were not available for healthy individuals or patients with SCA. The default Sim-Cancer population was used without any modification. The Sim-Cancer population was developed previously using this population as the baseline, and data from patients with solid tumours were included wherever possible. Details on physiological changes in cancer patients have been published earlier [50]. In summary, key changes in physiological parameters reflecting the effects of cancer include age distribution, weight and height relationships with age, reductions in GFR (37–258 mL/min) associated with elevated serum creatinine 40–149 µmol/L), a decrease in albumin, and an increase in AAG levels. The hydroxyurea absorption phase was then described using the advanced dissolution, absorption, and metabolism (ADAM) model to account for intestinal transporter kinetics with permeability across different segments of the gut being predicted from hydroxyurea physicochemical properties [38], as there are no published experimental values for hydroxyurea effective permeability in man (P_eff,man_), or for its intrinsic transcellular permeability, (P_trans,0_). The intrinsic clearance of an intestinal uptake transporter was optimised to recover oral data [11]. The input parameters used for the hydroxyurea PBPK model are summarised in Table 1.

The developed model was then verified using hydroxyurea in SCD patients using different dosing regimens. In this work, the default Sim-Healthy Volunteer was used to mimic the SCD clinical trial settings without any modification. Currently, a dedicated SCA population file is not available in the Simcyp Simulator. Moreover, an earlier study shows no significant differences in hydroxyurea PK between healthy and SCD adult women [31], although the sample size was small. Results from these simulations were compared to observed hydroxyurea PK in adults published in different clinical studies (Table 2).

The developed adult PBPK model was verified for its ability to predict hydroxyurea PK in paediatric SCA patients. This extrapolation was performed by using the default Sim-Paediatric population, that accounts for age-dependent physiology during development, including the maturation of the renal function [48], without any modification. The generic additional systemic clearance is scaled by default using allometric principles with a coefficient of 0.75 in the paediatric model, while the intestinal apical uptake CL_int,T_ was assumed to be as in adult, i.e., without any ontogeny.

Various virtual trials mimicking those reported in clinical studies (Table 2) were simulated, and the obtained PBPK results for hydroxyurea were compared to the clinically observed PK in SCD paediatric patients.

Subsequently, the verified adult PBPK model was extended to predict hydroxyurea PK in maternal plasma and breast milk by incorporating postpartum-dependent changes in maternal and milk physiology and interindividual distributions [33] using the default lactation model [34]. The lactation model uses the following equation (Equation (1)) to predict hydroxyurea transfer into breast milk by estimating the milk-to-plasma (M/P) ratio:

(1) $$M/P\;=\;{\mathrm{fu}}_{p}\cdot \frac{{\mathrm{fu}}_{p}^{\mathrm{un}}}{\left({\mathrm{fu}}_{\mathrm{mk}}^{\mathrm{un}}\cdot {\mathrm{fu}}_{\mathrm{mk}}\cdot \frac{1}{1\;+\;f_{\mathrm{fat}}\cdot \left({\mathrm{fu}}_{\mathrm{mk}}\cdot {\mathrm{Papp}}_{\mathrm{mk}}-1\right)}\right)}$$

In this equation, fu_p_ is the unbound fraction of hydroxyurea in maternal plasma, while ${\mathrm{fu}}_{p}^{\mathrm{un}}$ and ${\mathrm{fu}}_{\mathrm{mk}}^{\mathrm{un}}$ denote the unionised fractions of hydroxyurea in plasma and milk, calculated using the Henderson–Hasselbalch equations. The term fu_mk_ refers to the unbound fraction of hydroxyurea in skimmed milk. Since there are no measured values for fu_mk_, it can be predicted using Equation (2) [40]. The term f_fat_ represents the fractional volume of fat in milk, and Papp_mk_ represents the milk lipid-to-water partition coefficient, estimated using Equation (3) [40]:

(2) $${\mathrm{fu}}_{\mathrm{mk}}\;=\frac{{{\mathrm{fu}}_{p}}^{0.448}}{0.000694^{0.448}\;+\;{{\mathrm{fu}}_{p}}^{0.448}}$$

(3) $${\mathrm{Papp}}_{\mathrm{mk}}\;=10^{(-0.88+1.29\cdot \mathrm{logD}7.2)}$$

Here, logD7.2 is the apparent distribution coefficient at pH 7.2, estimated from hydroxyurea’s octanol-to-water partition coefficient (logP_o:w_).

The DID of hydroxyurea received through breast milk was calculated using the predicted average drug concentration in milk (C_avg,mk_) and the infant’s daily milk intake according to Equation (4):

(4) $$\mathrm{DID}\;\left(\mathrm{mg}/\mathrm{kg}/\mathrm{day}\right)\;=\;C_{\mathrm{avg},\mathrm{mk}}\;(\mathrm{mg}/L)\cdot \mathrm{daily}\;\mathrm{milk}\;\mathrm{intake}\;(L/\mathrm{kg}/\mathrm{day})$$

The daily milk intake varies with postpartum time (PpT) in months [33] according to Equations (5) and (6) in the lactation model:

If PpT < 1 month,

(5) $$\mathrm{Daily}\;\mathrm{milk}\;\mathrm{intake}\;(L/\mathrm{kg}/\mathrm{day})=\frac{0.181\cdot {\mathrm{PpT}}^{2.411}}{0.114^{2.411}\;+{\mathrm{PpT}}^{2.411}}$$

If PpT 1 to 12 months,

(6) $$\mathrm{Daily}\;\mathrm{milk}\;\mathrm{intake}\;(L/\mathrm{kg}/\mathrm{day})=0.004+(0.208-0.004)\;e^{(-0.15\cdot \mathrm{PpT})}\;$$

Infant body weight over the same age range (0–12 months postpartum) was predicted within the lactation model using equations (Equations (7) and (8)) that account for sex-specific growth trajectories.

For male infants,

(7) $$\mathrm{Body}\;\mathrm{weight}\;(\mathrm{kg})=\frac{9.50\cdot {\mathrm{PpT}}^{1.06}}{7.40\;+{\mathrm{PpT}}^{1.06}}+3.34\;$$

For female infants,

(8) $$\mathrm{Body}\;\mathrm{weight}\;(\mathrm{kg})=\frac{9.28\cdot {\mathrm{PpT}}^{1.05}}{8.60\;+{\mathrm{PpT}}^{1.05}}+3.28\;$$

Additionally, the relative infant’s daily dose (RID), relative to the mother’s daily dose adjusted for their body weight, was calculated according to Equation (9):

(9) $$\mathrm{RID}\;\left(\%\right)\;=\;\frac{\mathrm{DID}\;(\mathrm{mg}/\mathrm{kg}/\mathrm{day})}{\mathrm{Mother}\;\mathrm{daily}\;\mathrm{dose}\;(\mathrm{mg}/\mathrm{kg}/\mathrm{day})}\cdot 100$$

An RID greater than 10% is generally considered a threshold above which the medication may pose an increased risk during breastfeeding and warrants additional caution [32]. This threshold was therefore used as a benchmark for the safety interpretation of the simulated exposure levels.

Hydroxyurea PBPK model applications: Finally, different simulations were conducted to predict hydroxyurea exposure in breast milk in lactating women and to assess whether breastfeeding warrants significant exposure to breastfed children at different postnatal ages. To perform this analysis, the hydroxyurea level in breast milk was first predicted after two maternal daily dosing scenarios; the minimum dose (15 mg/kg/day) and the maximum tolerated dose (35 mg/kg/day) [55] to virtual lactating women (*n* = 200 per dose level). The corresponding daily infant dose (DID) and RIDs were calculated (Table 2). Simulated virtual breastfed children to these nursing mothers were

- Breastfed children aged 0.033, 0.23, 0.92, and 6 months (*n* = 200 per age group) who receive hydroxyurea only through breast milk.
- Breastfed children aged 6, 9, and 12 months (*n* = 200 per age group) who receive hydroxyurea through breast milk but also receive 20 mg/kg/day hydroxyurea therapy for SCA [27]. The combined daily dose (IDD + therapeutic dose) was then used as model input.

The exposure ratio in each of the paediatric population was calculated according to Equation (10), which considers age-dependent physiology in children:

(10) $$\mathrm{Exposure}\;\mathrm{ratio}\;\left(\%\right)\;=\;\frac{{\mathrm{AUC}}_{\mathrm{child}}}{{\mathrm{AUC}}_{\mathrm{mother}}\;}\cdot 100$$

Assessment criteria: Depending on data availability, the predicted PK profiles and/or parameters were compared with the various clinical observations reported in the literature. The PBPK model predictions were deemed successful if the observed PK profiles fell within the 5th to 95th percentile range of predictions and if the predicted PK parameters were within 0.5- to 2-fold of the observed values. The 5th–95th percentile prediction intervals provide an evidence-based plausible range of typical or expected plasma concentrations for the simulated virtual design considering the variability in the PBPK model (compound-, population-, and trial-dependent) parameters. They also aim to identify deviations, quantification of uncertainty, and more informed future study design and clinical decision-making.

## 5. Conclusions

Lactation PBPK modelling provides a valuable framework for evaluating the safety of breastfeeding during maternal hydroxyurea treatment. Simulations across a range of clinical scenarios indicate minimal (<10% of maternal dose) exposure to the breastfed infant. The estimation of PK-based exposure, along with uncertainty, and the identification of additional toxicology or clinical safety evidence required to assert that breastfeeding is “safe”, particularly during the first month postpartum, when predicted relative exposure is at its peak, are crucial considerations. In very young infants, targeted mitigation strategies such as pumping-and-dumping of milk after 3 h of maternal drug intake may be employed to ensure continued breastfeeding and maternal hydroxyurea use while minimising risks to the infant. A clinical validation of this model-based mitigation strategy is suggested. For older SCD infants who are already receiving hydroxyurea, the additional exposure associated with breastfeeding was shown to be negligible and is unlikely to contribute to adverse effects.

## Figures and Tables

**Table 1:** Overview of model parameters used in the adult hydroxyurea PBPK model.

	Parameter (Unit)	Value (Reference/Note)
Physicochemical properties and blood binding	Molecular weight (g/mol)	76.0
	logP	−1.8
	Compound type	Monoprotic Acid
	pKa	10.6
	B/P	1.0 [30]
	Binding protein (Plasma fu_p_)	Human serum albumin (0.77 [37])
Absorption	Absorption model	ADAM
	P_eff,man_ (×10^−4^ cm/s)	2.2 (predicted using MechPeff model)
	P_trans,0_ (×10^−6^ cm/s)	4.3 (predicted from logP_o:w_ using Method 1 [38])
	Intestinal apical uptake CL_int,T_ (µL/min)	6 (optimised to recover oral data [11])
Distribution	Distribution model	Full PBPK
	V_ss_ input type	Predicted (method 2 according to [39])
	Kp scalar	1.2 (optimised to recover [11])
Elimination	Clearance type	Enzyme kinetics
	CL_r_ (L/h)	4.2 (optimised to recover urinary data from [11])
	Additional systemic clearance (L/h)	4.8 (40% CV) (optimised to recover [11])
Lactation	Lactation model	Dynamic
	Milk fu_mk_	Predicted (according to Equation (2) [40])
	Papp_mk_	Predicted (according to Equation (3) [40])

Abbreviations: logP: neutral species octanol: buffer partition coefficient; pKa: acid dissociation constant at logarithmic scale; B/P: blood-to-plasma partition ratio; fu_p_: fraction unbound in plasma; P_eff,man_: jejunum effective permeability in man; P_trans,0_: intrinsic transcellular permeability; Kp scalar: a scalar applied to all predicted tissue-to-plasma partition (Kp) values; CL_r_: renal clearance; fu_mk_: fraction unbound in the skimmed milk; Papp_mk_: apparent partition coefficient for hydroxyurea between the lipids and water phases of the milk (see Section 4 for details).

**Table 2:** Overview of available clinical trial designs.

Study	Population	Age Range (yrs)	N (Female %)	Dosing Regimen
Rodriguez et al. [11]	Adult cancer	28–88	22 (30)	2000 mg 0.5 h IV
	Adult cancer	28–88	22 (30)	2000 mg oral
Duramed application 1997 [41]	Healthy adult	18–40	24 (0)	2000 mg SD PO (test)
	Healthy adult	18–40	24 (0)	2000 mg SD PO (reference)
De Forni et al. [42]	Healthy adult	18–55	12 (32)	500 mg SD PO
	Healthy adult	18–55	12 (32)	600 mg SD PO
	Healthy adult	18–55	12 (32)	600 mg SD PO
	Healthy adult	18–55	12 (32)	600 mg SD PO (Fed)
de Montalembert et al. [43]	Adult SCA	21–49	15 (50 *)	20.9 mg/kg QD PO (capsules)
	Adult SCA	21–49	15 (50 *)	20.9 mg/kg QD PO (tablets)
Pressiat et al. [16]	Adult SCA	23–79	51 (50)	15 mg/kg QD PO
Yan et al. [14]	Adult SCA	23–47	7 (43)	15 mg/kg SD PO
Wiczling et al. [18]	Paediatric SCA	5–17	21 (57)	20.3 mg/kg SD PO
de Montalembert et al. [43]	Paediatric SCA	4–19	11 (50 *)	21.9 mg/kg SD PO
Ware et al. [44]	Paediatric SCA	1.2–16.6	35 (87)	20 mg/kg SD PO
Estepp et al. [45]	Paediatric SCA	6–17	22 (50 *)	22.7 mg/kg SD PO liquid
	Paediatric SCA	6–17	22 (50 *)	22.6 mg/kg SD PO capsules
Nazon et al. [46]	Paediatric SCA	4–16	9 (56)	19 mg/kg SD PO
Rankine-Mullings et al. [17]	Paediatric SCA	0.5–17.99	32 (50)	25.8 mg/kg SD PO
	Paediatric SCA	0.5–1.99	6 (83)	15 mg/kg SD PO
	Paediatric SCA	2–5.99	16 (31)	15 mg/kg SD PO
	Paediatric SCA	6–17.99	10 (50)	15 mg/kg SD PO
Estepp et al. [47]	Paediatric SCA	8.8–14.1	7 (50 *)	17.9 mg/kg SD PO
	Paediatric SCA	7.9–10.6	52 (50 *)	23.8 mg/kg SD PO
Sylvester et al. [29]	A lactating woman with leukaemia	NA	1 (100)	500 mg T.I.D PO
Ware et al. [31]	Lactating women (14 healthy and 2 with SCA)	27–40	16 (100)	1000 mg QD PO
Marahatta et al. [30]	A lactating woman with SCA	31	1 (100)	1000 mg QD PO

* Sex distribution was not reported (50% female was assumed). NA: not available.

**Table 3:** Predicted versus observed pharmacokinetic parameter values of hydroxyurea, along with the corresponding predicted-to-observed (P/O) ratios, across different adult populations. Details of the study design can be found in Table 2.

Scheme		Oral Dose	AUC_inf_ (h·mg/L)			C_max_ (mg/L)			CL_po_ (L/h)			*fe* (%)		
			Obs.	Pred.	P/O	Obs.	Pred.	P/O	Obs.	Pred.	P/O	Obs.	Pred.	P/O
Rodriguez et al. [11]		2 g i.v.	270 ± 89	279 ± 82	1.0	76.5 ± 28.2	82.1 ± 15	1.1	6.4 ± 4.7	7.7 ± 2.3	1.2	35.8 ± 14	33.3 ± 6.7	1.0
		2 g	299 ± 83	266 ± 82	0.9	60.3 ± 18.3	49.1 ± 11	0.8	7.5 ± 3.3	8.2 ± 2.5	1.1	36.2 ± 7.6	33.8 + 7.0	0.9
Duramed [41]		2 g	215 ± 35	228 ± 57	1.1	50.5 ± 8.1	42.5 ± 7.3	0.8	9.3	9.7 ± 2.4	1.0	NA	47.9 ± 8.7	NA
		2 g	213 ± 39	228 ± 57	1.1	48.1 ± 14.1	42.5 ± 7.3	0.9	9.4	9.7 ± 2.4	1.0	NA	47.9 ± 8.7	NA
De Forni et al. [42]		500 mg	56 (46–71)	54.3 ± 14.2	1.0	13.1 (10.6–18.8)	11.0 ± 2.4	0.8	8.9	9.5 ± 2.5	1.1	NA	48.8 ± 9.2	NA
		600 mg	70 (58–80)	65.6 ± 16.3	0.9	16.2 (11.5–20.3)	13.0 ± 2.9	0.8	8.6	9.4 ± 2.3	1.1	NA	49.4 ± 9.0	NA
		600 mg	70 (59–85)	65.6 ± 16.3	0.9	15 (9.7–22)	13.0 ± 2.9	0.9	8.5	9.4 ± 2.3	1.1	NA	49.4 ± 9.0	NA
		600 mg	65 (47–81)	65.1 ± 17.0	1.0	10.5 (7.2–15)	10.3 ± 3.0	1.0	9.2	9.5 ± 2.5	1.0	NA	48.8 ± 9.2	NA
de Montalembert et al. [43]		20.9 mg/kg	128.4 ± 39	145 ± 27	1.1	26.5 ± 13.9	33.2 ± 4.6	1.2	9.4 ± 2.7	9.1 ± 2.2	1.0	NA	47.4 ± 10.0	NA
Pressiat et al. [16]		15 mg/kg	86.9 ± 28 ^$^	117 ± 40 ^$^	1.3	20.7 ± 12.5	25.8 ± 8.2	1.2	10.1 ± 4.1	9.8 ± 2.5	1.0	35.8 ± 10.2	47.8 ± 9.6	1.3
Yan et al. [14]		15 mg/kg	82.5 ± 15.5	124 ± 18.6	1.5	28.3 ± 11.0	29.3 ± 4.7	1.0	13.9 ± 3.7	10 ± 2.5	0.7	37.7 ± 18.0	49.5 ± 9.4	1.3
Ware et al. [31]	Plasma	1000 mg	74.8 ± 29 ^$^	102 ± 29.1 ^$^	1.4	19.5 ± 8.9	19.9 ± 4.3	1.0	10.0 ± 2.4	10.5 ± 2.7	1.1	54.2 ± 17.8	52.4 ± 12.6	1.0
	Milk		62.5 ± 26 ^$^	79.0 ± 21.1 ^$^	1.3	12.9 ± 5.0	15.4 ± 3.4	1.2	NA	NA	NA	NA	NA	NA

Values given as mean ± SD. AUC_inf_: extrapolated area under the curve; C_max_: maximum concentration in plasma; CL_po_: total oral clearance; *fe*: fraction of drug excreted unchanged in urine. ^$^ AUC_0–24_ reported; predicted values are also AUC_0–24_ for consistency. NA: not applicable.

**Table 4:** Comparison of predicted versus observed hydroxyurea PK parameter values, including the predicted-to-observed (P/O) ratios in paediatric populations with SCD.

Study	Dose (mg/kg)	AUC_inf_ (h·mg/L)			C_max_ (mg/L)			CL (L/h)			T_max_ (h)		
		Obs.	Pred.	P/O	Obs.	Pred.	P/O	Obs.	Pred.	P/O	Obs.	Pred.	P/O
Wiczling et al. [18]	20	102.0 ± 30.0	123.3 ± 45.7	1.21	33.2 ± 13.2	28.1 ± 10.6	0.85	8.0 ± 3.7	6.6 ± 3.1	0.83	0.7 ± 0.4	1.2 ± 1.2	1.71
De Montalebert et al. [43] *	21.9	115.8 ± 45.2	133.7 ± 29.8	1.15	24.5 ± 11.8	30.8 ± 7.3	1.26	4.5 ± 5.3	7.0 ± 3.7	1.56	0.8	1.0 ± 0.7	1.25
Ware et al. [44]	20	93.0 ± 23.4	119.9 ± 37.3	1.29	26.1 ± 6.8	27.3 ± 8.5	1.05	6.9 ± 3.2	5.6 ± 3.1	0.81	0.8 ± 0.5	1.2 ± 0.7	1.50
Estepp et al. [45]	22.7	116.3 ± 30.0	136.6 ± 37.8	1.17	33.6 ± 8.2	31.1 ± 9.0	0.93	8.2 ± 1.2	6.6 ± 3.0	0.80	1.0 ± 0.5	1.2 ± 0.7	1.20
	22.6	111.9 ± 29.4	137.8 ± 38.1	1.23	34.0 ± 8.7	31.4 ± 9.1	0.92	8.6 ± 1.6	6.6 ± 3.0	0.77	0.7 ± 0.5	1.2 ± 0.7	1.71
Nazon et al. [46]	19	81.3 ± 25.2 ^$^	89.3 ± 13.7 ^$^	1.10	24.1 ± 9.1	22.4 ± 4.5	0.93	NA	5.7 ± 2.7	NA	1.5 ± 0.6	1.7 ± 0.6	1.13
Rankine-Mullings et al. [17]	15	62.5 ± 9.0	76.1 ± 17.7	1.22	12.8 ± 1.4	15.8 ± 3.5	1.23	NA	2.3 ± 0.9	NA	1.3 ± 0.3	1.6 ± 0.6	1.23
	15	62.9 ± 7.4	81.5 ± 17.1	1.30	13.1 ± 1.9	16.5 ± 3.4	1.36	NA	3.4 ± 1.3	NA	1.2 ± 0.5	1.6 ± 0.6	1.33
	15	68.9 ± 11.6	95.0 ± 20.4	1.38	13.4 ± 1.7	17.6 ± 3.6	1.31	NA	7.2 ± 3.4	NA	1.3 ± 0.4	1.7 ± 0.6	1.31
Estepp et al. [47]	17.9	105.1 ± 26.2	108.3 ± 31.1	1.03	35.5 ± 14.6	24.7 ± 7.8	0.70	6.9 ± 1.2	6.8 ± 2.7	0.99	0.9 ± 0.6	1.2 ± 0.7	1.33
	23.8	107.3 ± 27.5	135.3 ± 38.6	1.26	33.8 ± 9.4	32.1 ± 10.1	0.95	7.2 ± 1.9	5.6 ± 2.0	0.78	0.8 ± 0.5	1.2 ± 0.7	1.50

Study design details are provided in Table 2. AUC_inf_, area under the plasma concentration versus time curve extrapolated to infinity; C_max_, maximum concentration in plasma; T_max_, time of maximum concentration; SD, standard deviation. * T_max_ reported and (for consistency) predicted values are medians. ^$^ Reported and predicted values are AUC_0–6h_. CL, total clearance after oral administration; NA: not applicable. Values given as mean ± SD except were specified.

**Table 5:** Model application scenarios for neonates and infants after maternal exposure to either low (15 mg/kg) or high (35 mg/kg) daily hydroxyurea doses at different postnatal age assuming the breastfed children are either healthy or receiving additional therapy for SCD.

Mother’s Dose	Postpartum Age (Months)	Maternal AUC_tau_ (h·mg/L)	Milk AUC_tau_ (h·mg/L)	Milk AUC_3h_ (h·mg/L)	M/P Ratio	DID (mg/kg/day)	RID (%)	Paediatric SCD Dose (mg/kg)	Total Paediatric Dose (mg/kg/day)	Paediatric AUC_tau_ (h·mg/L)	Exposure Ratio
15 mg/kg	0.033	94 ± 20	79 ± 17	46 ± 5.5	0.83	0.09 ± 0.03	0.6 ± 0.2	0	0.09	0.50 ± 0.20	0.53%
	0.23	97 ± 20	80 ± 16	45 ± 5.5	0.83	1.60 ± 0.50	10.9 ± 3.2	0	1.6	8.3 ± 2.8	8.6%
	0.92	103 ± 19	82 ± 16	46 ±5.4	0.80	1.90 ± 0.60	13.0 ± 3.9	0	1.9	9.6 ± 3.0	9.3%
	6	103 ± 19	80 ± 16	45 ± 5.6	0.78	0.93 ± 0.33	6.2 ± 1.9	0	0.93	4.40 ± 1.3	4.3%
								20	20.93	102 ± 29.5	99%
	9	103 ± 20	80 ± 16	45 ± 5.6	0.77	0.60 ± 0.20	4.0 ± 1.2	0	0.60	2.93 ± 0.84	2.8%
								20	20.6	101 ± 29	98%
	12	103 ± 19	80 ± 16	45 ± 5.4	0.77	0.40 ± 0.10	2.6 ± 0.8	6	0.40	1.96 ± 0.56	1.9%
								20	20.4	100.0 ± 28.4	97%
35 mg/kg	0.033	220 ± 47	185 ± 39	106 ± 13	0.83	0.2 ± 0.1	0.6 ± 0.2	0	0.2	1.20 ± 0.5	0.55%
	0.23	226 ± 46	187 ± 38	106 ± 13	0.83	3.8 ± 1.1	10.9 ± 3.2	0	3.8	19.7 ± 6.6	8.72%
	0.92	240 ± 45	192 ± 38	107 ± 13	0.80	4.5 ± 1.4	13.0 ± 3.9	0	4.5	22.8 ± 7.0	9.5%
	6	240 ± 45	187 ± 38	106 ± 13	0.78	2.2 ± 0.7	6.2 ± 1.9	0	2.2	10.8 ± 3.1	4.5%
								20	22.2	108 ± 31	45%
	9	240 ± 46	186 ± 38	105 ± 13	0.77	1.4 ± 0.4	4.0 ± 1.2	0	1.40	6.94 ± 1.93	2.9%
								20	21.4	105 ± 30	44%
	12	241 ± 46	186 ± 38	105 ± 13	0.77	0.92 ± 0.3	2.6 ± 0.8	0	0.92	4.51 ± 1.28	1.9%
								20	20.92	103 ± 29	43%

Values given as mean± SD. M/P: predicted milk-to-plasma ratio; DID: model prediction for the daily infant dose; RID: relative infant dose; total paediatric dose = IDD + SCD dose. Exposure ratio = (AUC_paed_/AUC_maternal_).

## Data Availability

The raw data supporting the conclusions of this article will be made available by the authors on request.

## Abbreviations

The following abbreviations are used in this manuscript:

DID	Daily Infant Dose
RID	Relative Infant Dose
PpT	Postpartum Time

## Appendix A

**Table A1:** Sensitivity analysis of model output to different values of intrinsic clearance assigned to the gut uptake transporter (CL_int,T_) in healthy adult and paediatric populations. Data shown as mean ± SD (N = 400 virtual subjects in each scenario).

Gut Uptake Transport CL_int,T_ (uL/min)		Healthy Adult (Observed PK from Duramed [41])			Paediatric (0.5–2.0 Years) (Observed PK: Rankine-Mullings et al. [17])		
		AUC_inf_ (h·mg/L)	T_max_ (h)	C_max_ (mg/L)	AUC_inf_ (h·mg/L)	T_max_ (h)	C_max_ (mg/L)
	Observed PK	213 ± 39	0.84 ± 1.15	48.1 ± 14.1	62.5 ± 9.0	1.3 ± 0.3	12.8 ± 1.4
0	Predicted	214 ± 54	1.71 ± 0.39	29.8 ± 5.5	74.5 ± 17.6	2.06 ± 0.58	13.9 ± 2.8
0.3	Predicted	228 ± 57	1.11 ± 0.28	40.5 ± 6.5	76.1 ± 17.7	1.82 ± 0.53	15.6 ± 3.3
0.6	Predicted	228 ± 57	0.98 ± 0.28	41.4 ± 6.7	76.1 ± 17.7	1.74 ± 0.54	15.8 ± 3.4
3	Predicted	228 ± 57	0.8 ± 0.29	42.4 ± 7.2	76.1 ± 17.7	1.62 ± 0.56	15.8 ± 3.4
6	Predicted	228 ± 57	0.74 ± 0.3	42.5 ± 7.3	76.1 ± 17.7	1.59 ± 0.56	15.8 ± 3.5
12	Predicted	228 ± 57	0.71 ± 0.31	42.6 ± 7.5	76.1 ± 17.7	1.56 ± 0.57	15.8 ± 3.5
30	Predicted	228 ± 57	0.67 ± 0.32	42.7 ± 7.6	76.1 ± 17.7	1.53 ± 0.57	15.8 ± 3.5
60	Predicted	228 ± 57	0.66 ± 0.32	42.7 ± 7.6	76.1 ± 17.7	1.51 ± 0.58	15.8 ± 3.5

**Table A2:** PBPK model-derived milk AUC of remaining daily infant dose (DID) after pumping-and-dumping milk collected within 2, 3, and 4 h of maternal hydroxyurea administration.

Postpartum (Months)	0 Hour (No Discard)		0–2 h			0–3 h			0–4 h		
	AUC_24h_ (h·mg/L)	DID (mg/k)	AUC_2–24h_ (h·mg/L)	DID (mg/kg)	DID * (%)	AUC_3–24h_ (h·mg/L)	DID (mg/kg)	DID * (%)	AUC_4–24h_ (h·mg/L)	DID (mg/kg)	DID * (%)
15 mg/kg											
0.03	79	0.09	45.9	0.05	58%	33	0.04	42%	24.5	0.03	31%
0.23	80	1.6	47	0.94	59%	35	0.70	44%	25.6	0.51	32%
0.92	82	1.9	48.6	1.13	59%	36	0.83	44%	27	0.63	33%
6	80	0.93	46.9	0.55	59%	35	0.41	44%	25.7	0.30	32%
9	80	0.6	47	0.35	59%	35	0.26	44%	25.9	0.19	32%
12	80	0.4	47.1	0.24	59%	35	0.18	44%	25.9	0.13	32%
35 mg/kg											
0.03	185	0.2	107.8	0.12	58%	79	0.09	43%	58	0.06	31%
0.23	187	3.8	109.9	2.23	59%	81	1.65	43%	60	1.22	32%
0.92	192	4.5	114	2.67	59%	85	1.99	44%	64	1.50	33%
6	187	2.2	109.7	1.29	59%	81	0.95	43%	60	0.71	32%
9	186	1.4	109.1	0.82	59%	81	0.61	44%	60	0.45	32%
12	186	0.92	109.3	0.54	59%	81	0.40	44%	60	0.30	32%

* The DID as a proportion of the no discarding scenario.
